# Thermally modulated biomolecule transport through nanoconfined channels

**DOI:** 10.1186/s11671-015-0889-0

**Published:** 2015-04-25

**Authors:** Lei Liu, Lizhong Zhu

**Affiliations:** Jiangsu Key Laboratory for Design and Manufacture of Micro-Nano Biomedical Instruments, School of Mechanics, Southeast University, Si Pai Lou 2#, Nanjing, 210096 People’s Republic of China; Suzhou Key Laboratory for Design and Manufacture of Micro-Nano Biomedical Instruments, Suzhou Research Institute of Southeast University, Linquan street 399#, Suzhou, 215123 People’s Republic of China

**Keywords:** Nanopore arrays, Biosensing, Transporting property

## Abstract

In this work, a nanofluidic device containing both a feed cell and a permeation cell linked by nanopore arrays has been fabricated, which is employed to investigate thermally controlled biomolecular transporting properties through confined nanochannels. The ionic currents modulated by the translocations of goat antibody to human immunoglobulin G (IgG) or bovine serum albumin (BSA) are recorded and analyzed. The results suggest that the modulation effect decreases with the electrolyte concentration increasing, while the effects generated by IgG translocation are more significant than that generated by BSA translocation. More importantly, there is a maximum decreasing value in each modulated current curve with biomolecule concentration increasing for thermally induced intermolecular collision. Furthermore, the turning point for the maximum shifts to lower biomolecule concentrations with the system temperature rising (from 4°C to 45°C), and it is mainly determined by the temperature in the feed cell if the temperature difference exists in the two separated cells. These findings are expected to be valuable for the future design of novel sensing device based on nanopore and/or nanopore arrays.

## Background

In the past years, nanopore-based device is attracting more and more attentions for its potential applications in selective molecular separation [1,2], biosensing [3,4], energy storage [5,6], controlled release [7,8], drug delivery [9,10], nanofluidics, and nanoelectronics [11-14]. It is generally believed that the premise of this method is how to obtain proper nanopores and how to design effective fluidic device. Till now, natural nanopores in biomembranes [15,16] and artificial nanopores in inorganic films [17-19] are two major types of pores used in this area, while nanopores in polymer membranes obtained by track-etching method also can provide other possible choices [20-23]. Since the transport of mass and charge in the confined nanochannels, novel phenomena which cannot be observed in the bulk solutions will occur [24-26]. As we known, the flow of water and the molecular mobility in confined spaces is much faster than that in bulk case [27-29]. These phenomena indicate that the controlled transport of mass and charge through confined spaces is a fundamental process of interest in biology, physics, and chemistry, which have been regarded as an emerging field with major impact on bioanalysis and fundamental understanding of nanoscale interactions down to single molecule level [30-32]. Therefore, molecule or ion transport through nanopores is a key scientific issue for the fundamental understandings of the physical nature of analyte translocation. Although many important progresses have been established in this area, bridging theoretical calculations and experimental results conformably and ending up with an in-depth understanding of all problems is not an easy task. In this work, nanopore array-based sensing device has been fabricated by using PC ultrafiltration membrane, which is employed to investigate the thermally controlled biomolecular transporting properties in confined nanochannels.

## Methods

**Figure 1:**
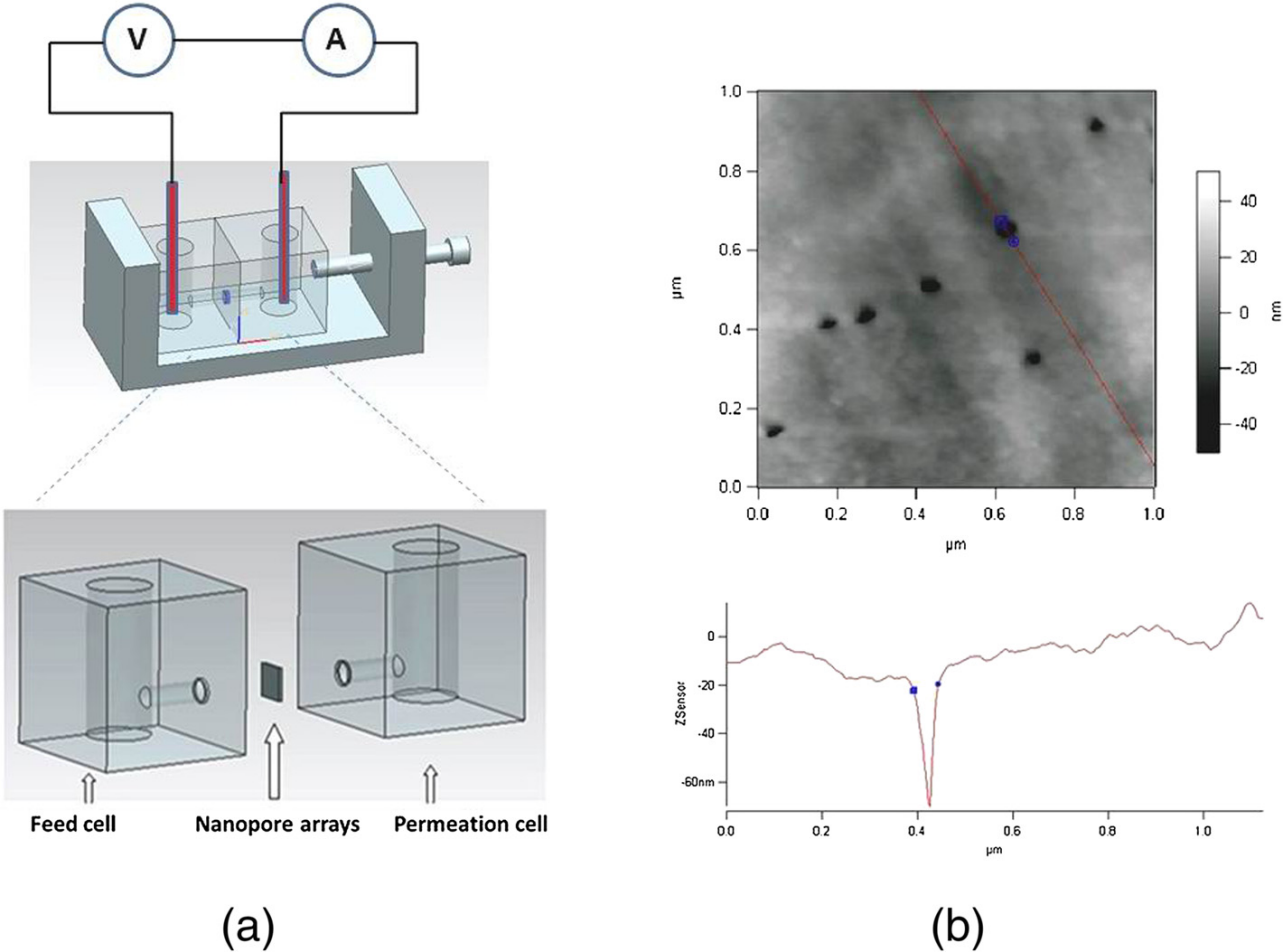
Nanofluidic device and the AFM characterization for 50-nm pore. **(a)** Prototype nanofluidic device based on nanopore arrays. The left cell in which biomolecules are added is the feed cell, and the right cell is the permeation cell; **(b)** AFM characterization for 50-nm pore arrays.

Goat antibody to human immunoglobulin G (IgG) and bovine serum albumin (BSA) (bought from Nanjing Boquan Technology Co., Ltd., Nanjing, China) were used as analytes. KCl (analytical grade) solutions were employed as electrolyte. As illustrated in Figure 1a, nanofluidic device was made up of two separated cells linked by a chip containing nanopore arrays (pore diameter: 50 nm used for IgG and 15 nm used for BSA, the AFM characterization for 50-nm pore is shown in Figure 1b). The temperature in the feed cell and the permeation cell were controlled at 4°C, 22°C, and 45°C. The transmembrane ionic current under the applied voltage of 2.5 V was measured by Keithley 2000 multimeter (Keithley Instruments, Inc., Cleveland, OH, USA).

**Figure 2:**
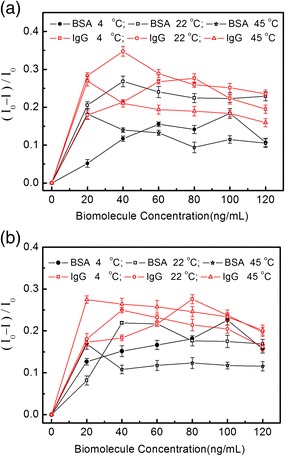
The value of (*I*_0_ − *I*)/*I*_0_ modulated by IgG and BSA in 0.01 mol/L **(a)** and 1.0 mol/L **(b)** KCl solution. The temperatures are controlled at 4°C, 22°C, and 45°C, respectively. (*I*_0_ − *I*)/*I*_0_ is defined as the relative variance ratio reflected the modulated current changing tendency, where *I*_0_ and *I* stand for the ionic current without and with biomolecules modulation.

Obviously, ions (K^+^ and Cl^−^) in the solution moved directionally through nanopores result in the basic ionic current (*I*_0_), while IgG or BSA molecules driven to pass through nanopores generate the modulated ionic currents (*I*). Generally, the change in the ionic current will be mainly affected by biomolecules’ translocation. Once or BSA molecules enter nanopores, the volumes in the nanopores are partially occupied, which will prevent certain amounts of K^+^ and Cl^−^ from passing through the confined channel, which will decrease the background ionic current (*I*_0_). At the same time, the surface charge of IgG molecule will also contribute to the increase of the total ionic current when it passes through nanopore. The final current changes will be determined by the combined effects. Generally, the modulated ionic current (*I*) is smaller than the basic ionic current (*I*_0_) if the concentration of the electrolyte solution is not too low. So the relative changing tendency of the modulated current can be reflected by the value of (*I*_0_ − *I*)/*I*_0_. Figure 2a,b shows the values of (*I*_0_ − *I*)/*I*_0_ modulated by IgG and BSA in the electrolyte concentration of 0.01 and 1.00 mol/L, respectively, which are obtained at 4°C, 22°C, and 45°C. Comparing Figure 2a,b the modulated effects generated by biomolecules’ translocation decrease with the electrolyte concentration increasing because of the background ionic current increasing, and the modulated effects caused by IgG (stocks diameter: 25 nm) are more significant than those caused by BSA (stocks diameter: 3.5 nm). More importantly, there exists a maximum decreasing value in each curve, which means the biggest place holding effect by biomolecules. The turning point concentration values corresponding to the maximum for IgG are 80 ng/mL (4°C), 40 ng/mL (22°C), and 20 ng/mL (45°C), respectively. Those values for BSA are 100 ng/mL (4°C), 40 ng/mL (22°C), and 20 ng/mL (45°C), respectively. Although there are slight differences in the detailed value for BSA and IgG, the changing tendencies for the two biomolecules are similar. It should be pointed out that the turning point for the modulated current curve with biomolecule concentration is the same with the turning point of biomolecule concentration corresponding to the maximum value of (*I*_0_ − *I*)/*I*_0_ for each case in our experiments.

## Results and discussion

**Figure 3:**
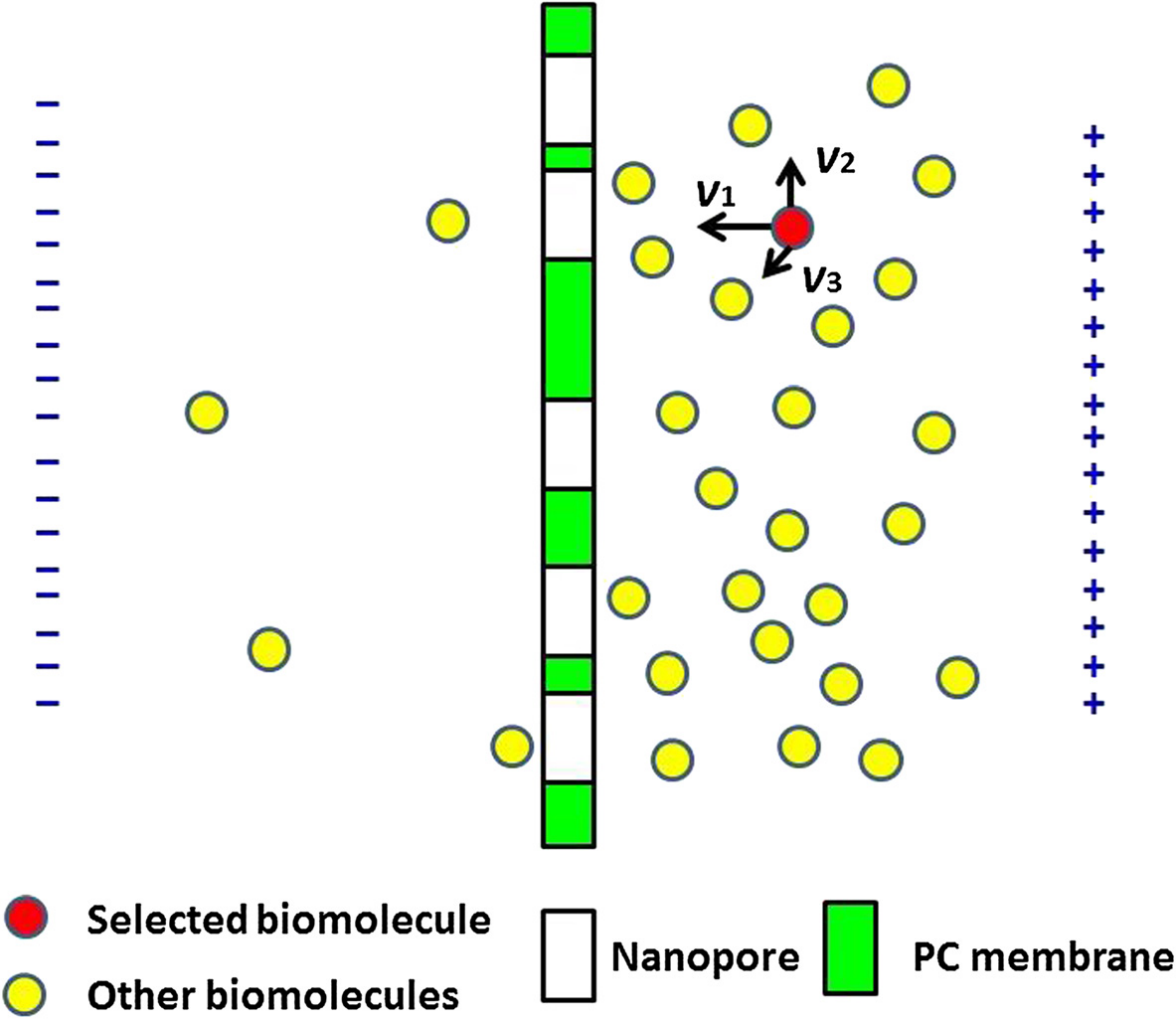
Schematic picture of a single biomolecule passing through nanopore arrays.

Figure 3 illustrates a single biomolecule (red ball) near a nanopore, and its sweeping volume in a period of time (*δt*) can be expressed as *πd*^*2*^*vδt* (*v*: velocity which containing the axial velocity *v*_1_ and the velocities in the other two mutually perpendicular directions represented as *v*_2_ and *v*_3_; *d:* stocks diameter of biomolecules). Its total number of collisions (*Z*) with other biomolecules in unit time can be expressed as *Z = πd*^*2*^*vn* (*n*: number of other biomolecules in unit volume). Obviously, *v*_2_ and *v*_3_ contribute little to the biomolecule translocation. For an upcoming captured biomolecule near nanopore, keeping the speed of *v*_1_ and continuing to move toward nanopore means translocation, while collisions will reduce its probability of translocation. Therefore, the increasing biomolecule concentration corresponds to the bigger number of molecules with potential translocation; while more biomolecules will gather near nanopores, the total number of collisions will also increase. The former raises the translocation probability, and the latter decreases the capture probability. Under the joint influences, there must exists a maximum value for IgG translocation with its concentration increasing.

As we known, temperature increasing means the larger internal energy, which can result in bigger moving intensity of biomolecules and ions. For one single biomolecule in the solution, its kinetic energy can be described as:

1 $$ E={E}_V+{E}_T=\frac{1}{2}m{v}^2=\frac{1}{2}m{v}_1^2+\frac{3}{2}{K}_BT $$

in which $\frac{1}{2}m{v}_1^2$ is mainly determined by electric field and $\frac{3}{2}{K}_BT$ is determined by temperature. Based on Equation (1), the total number of collisions (*Z*) in unit time can be rewritten as:

2 $$ Z=\pi {d}^2n\sqrt{v_1^2+\frac{3{K}_BT}{m}} $$

**Figure 4:**
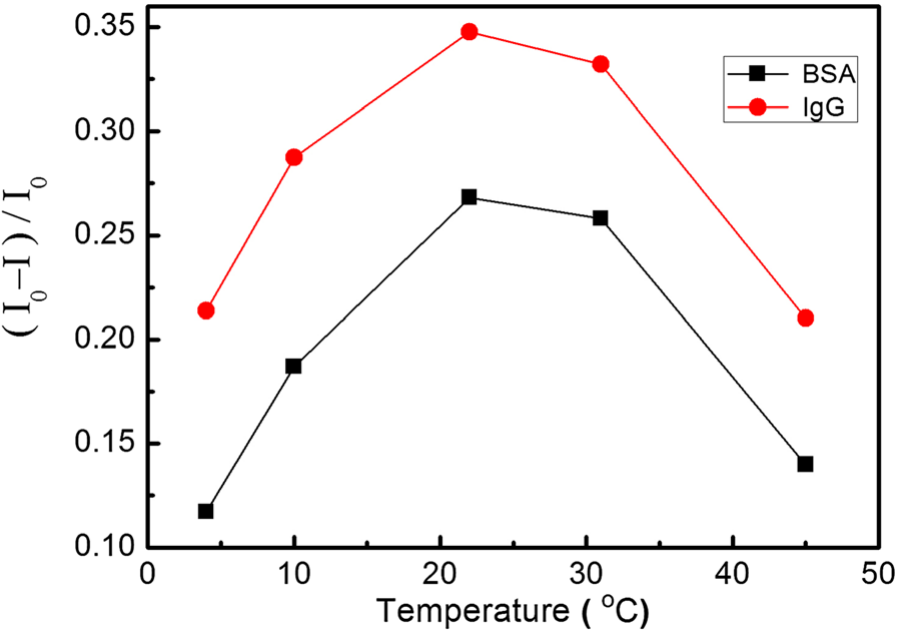
The value of (*I*_0_ − *I*)/*I*_0_ modulated by temperature. The electrolyte concentration is fixed at 0.01 mol/L, and protein concentration is fixed at 40 ng/L.

Furthermore, pH value of the solution will decrease slightly with the temperature rising. In our experiment, pH value decreases from 7.35 to 6.75 when the temperature increases from 4°C to 45°C. These changes in pH value can increase the charged amounts of biomolecule; therefore, higher temperature will result in bigger *v*_1_ under the same accelerating voltage. With temperature increasing, more biomolecules will aggregate near nanopores, and at the same time, the total number of collisions (*Z*) in unit time will increase. The effect caused by temperature increasing is determined by the joint action of the above two factors, which can be demonstrated in Figure 4. As shown in Figure 4, for both IgG and BSA, the turning point of biomolecule concentration corresponding to the maximum effect decreases with the temperature rising. Temperature changes will affect the intermolecular collisions, which will change the number of translocation. For two kinds of biomolecules, when the temperature is low, increasing temperature could lead to increasing thermal motion of protein molecules, which can promote protein migration; when the temperature continues to rise, when the molecular collision greatly increases, translocation current will decrease. Consequently, higher temperature will make earlier arrival to the saturation of biomolecules’ translocation, which results in the turning point for the maximum shifting to lower concentration for both IgG and BSA.

**Figure 5:**
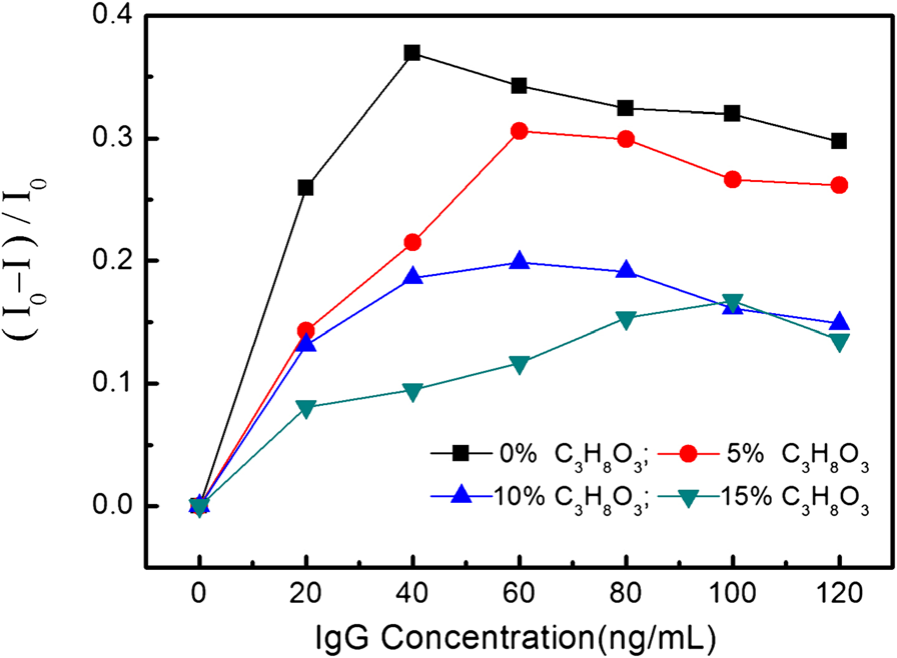
The value of (*I*_0_ − *I*)/*I*_0_ modulated by the viscosity of solution. Biomolecule: IgG, KCl: 0.1 mol/L, temperature: 22°C.

According to the above discussion, temperature affects the translocation of mainly through the speed of biomolecules and molecular collision between biomolecules. The number of collisions (*Z*) in local space can be calculated by Equation (2). In order to understand the influence on the translocation by the speed of biomolecules, similar experiments have been carried out (biomolecule: IgG, KCl: 0.1 mol/L, temperature: 22°C), and the difference is certain volume percent of glycerol which was added in the solution. Glycerol can change the viscosity of the solution and influence the speed of biomolecules. Of course, glycerol will also influence the basic ionic current; in our experiment, the detected basic currents are 44.7, 42.8, 40.3, and 35.9 μA when the volume percent of glycerol is 0%, 5%, 10%, and 15%, respectively. From the results shown in Figure 5, it indicates that the turning point for the maximum value of (*I*_0_ − *I*)/*I*_0_ shifts to bigger biomolecule concentrations, which mainly determined by the IgG speed decrease resulted from the viscosity increase of the solution.

**Figure 6:**
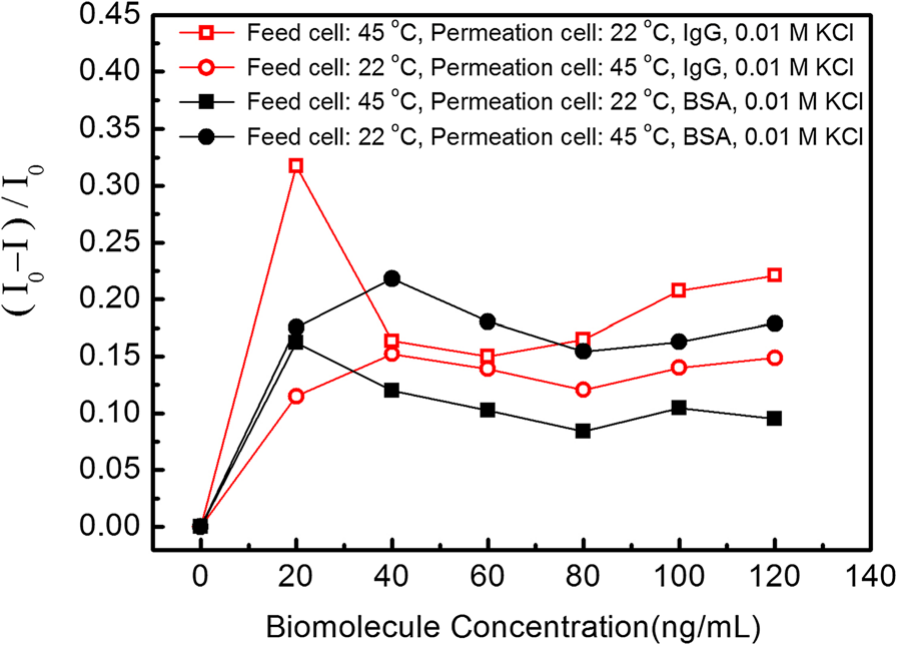
The value of (*I*_0_ − *I*)/*I*_0_ modulated by IgG and BSA in 0.01 mol/L KCl solution. The temperatures in feed cell and permeation cell are controlled at 45°C or 22°C, respectively. (*I*_0_ − *I*)/*I*_0_ is defined as the relative variance ratio reflected the modulated current changing tendency, where *I*_0_ and *I* stand for the ionic current without and with IgG molecules modulation.

In above discussion, the capture probability of IgG molecule which mainly influences biomolecule translocations and ionic currents is discussed in the region nearby nanopore on the side of feed cell. Once IgG or BSA molecule is captured by nanopore, it will move to the permeation cell under the strong voltage drop and then leave nanopore on the side of permeation cell rapidly after its translocation. So the turning point for the maximum (*I*_0_ − *I*)/*I*_0_ value is mainly defined by the temperature in the feed cell, which is supported by the experimental results obtained in temperature difference conditions. As shown in Figure 6, if the temperatures are controlled at 45°C in feed cell and 22°C in permeation cell, the turning point for IgG and BSA is similar to the case with the temperatures being controlled at 45°C in both cells. Conversely, if the temperatures are controlled at 22°C in feed cell and 45°C in permeation cell, the turning point for IgG and BSA is similar to the case with the temperatures being controlled at 22°C in both cells.

## Conclusions

In summary, the thermally controlled biomolecular transport properties have been investigated by a nanofluidic device with a feed cell and a permeation cell linked by a chip containing nanopores arrays. The results indicate that there exists a maximum decreasing value in each modulated current curve with biomolecule concentration increasing because of the thermally induced intermolecular collision, which is mainly influenced by the temperature in feed cell.
